# Are dominant plant species more susceptible to leaf‐mining insects? A case study at Saihanwula Nature Reserve, China

**DOI:** 10.1002/ece3.4284

**Published:** 2018-07-09

**Authors:** Xiaohua Dai, Chengpeng Long, Jiasheng Xu, Qingyun Guo, Wei Zhang, Zhihong Zhang, Bater

**Affiliations:** ^1^ Leafminer Group School of Life and Environmental Sciences Gannan Normal University Ganzhou China; ^2^ National Navel Orange Engineering Research Center Ganzhou China; ^3^ Saihanwula National Nature Reserve Administration Daban China

**Keywords:** apparency, importance value, Leafminer, phylogenetic generalized linear mixed model (PGLMM), species–area relationship

## Abstract

Dominant species significantly affect interspecific relationships, community structure, and ecosystem function. In the field, dominant species are often identified by their high importance values. Selective foraging on dominant species is a common phenomenon in ecology. Our hypothesis is that dominant plant groups with high importance values are more susceptible to leaf‐mining insects at the regional level. Here, we used the Saihanwula National Nature Reserve as a case study to examine the presence–absence patterns of leaf‐mining insects on different plants in a forest‐grassland ecotone in Northeast China. We identified the following patterns: (1) After phylogenetic correction, plants with high importance values are more likely to host leafminers at the species, genus, or family level. (2) Other factors including phylogenetic isolation, life form, water ecotype, and phytogeographical type of plants have different influences on the relationship between plant dominance and leafminer presence. In summary, the importance value is a valid predictor of the presence of consumers, even when we consider the effects of plant phylogeny and other plant attributes. Dominant plant groups are large and susceptible targets of leaf‐mining insects. The consistent leaf‐mining distribution pattern across different countries, vegetation types, and plant taxa can be explained by the “species‐area relationship” or the “plant apparency hypothesis.”

## 1 INTRODUCTION

Not all species play equal roles in a given community or ecosystem. Dominant species are the small number of species that significantly affect other species (McNaughton & Wolf, 1970; Whittaker, 1965). Due to their high biomass, large size, high productivity, and other traits (Bouchenak‐Khelladi, Slingsby, Verboom, & Bond, 2014; Collins & Duffy, 2016), they can change environmental conditions and resource availability and thus shape community structure (Frieswyk, Johnston, & Zedler, 2007; Okullo, Greve, & Moe, 2013), community diversity (Kunte, 2008; Okullo et al., 2013), community phylogeny (Chalmandrier, Münkemüller, Lavergne, & Thuiller, 2015), trophic structure (Miller, Brodeur, Rau, & Omori, 2010), and ecosystem functions (Behera et al., 2017; Furey, Tecco, Perez‐Harguindeguy, Giorgis, & Grossi, 2014; Grime, 1998; Mokany, Ash, & Roxburgh, 2008; Seabloom et al., 2015). Both dominant species and keystone species are functionally important, but keystone species are much less abundant (Christianou & Ebenman, 2005; Hurlbert, 1997; Mouquet, Gravel, Massol, & Calcagno, 2013; Power et al., 1996). Therefore, dominant species with high abundance might contribute more to an ecosystem (Perry, 2010). Furthermore, dominance can be species‐, morphospecies‐, functional group‐, or plant life form‐based (Engemann et al., 2016; Gonmadje et al., 2011).

Both abiotic and biotic factors can be used to identify dominant species (Frieswyk et al., 2007; Koike, 2001; Yu et al., 2015). Although many quantitative traits, such as density, cover, or biomass, can be used to measure community dominance, the use of combinations of multiple variables may be more appropriate (Guo & Rundel, 1997). As the importance value encompasses cover, frequency, abundance, and, occasionally, diversity (Curtis & McIntosh, 1951; Gonmadje et al., 2011; Mori, Boom, de Carvalino, & dos Santos, 1983), it is expected to be a good indicator of dominance or apparency (Brandt, Zimmermann, Hensen, Mariscal Castro, & Rist, 2012; Dahdouh‐Guebas, Koedam, Satyanarayana, & Cannicci, 2011; Dahdouh‐Guebas, Verheyden, De Genst, Hettiarachchi, & Koedam, 2000; Dai, Zhang, Xu, Duffy, & Guo, 2017; Gonçalves, Albuquerque, & de Medeiros, 2016; Guèze et al., 2014; Guo, Li, Liu, & Zhou, 2012; Hu, Su, Li, Li, & Ke, 2015; Smith & Smith, 2001; Soldati, de Medeiros, Duque‐Brasil, Coelho, & Albuquerque, 2017; Thomas, Vandebroek, & Van Damme, 2009). Importance values can be applied to detect dominant species in different communities, especially along ecological gradients (Greig‐Smith, 1983; Henkel, Chambers, & Baker, 2016; Kent, 2012). In practice, dominants are often defined as those plant groups with high importance values (Gonmadje et al., 2011; Khairil, Juliana, Nizam, Wan Juliana, & Nizam, 2014; Schmook, 2010; Wu, Shinzato, Kudo, Ishigaki, & Aramoto, 2008).

The degree or level of species dominance can be influenced by both physical and biological factors. Environmental conditions can directly or indirectly shape dominance patterns in biotic communities (Endress, Naylor, Parks, & Radosevich, 2007; Poulos, Taylor, & Beaty, 2007; Schweiger & Beierkuhnlein, 2014). Strengthened interspecific competition between dominants and subordinates influences the fate of the latter group, as weak competition permits an inferior species to persist for a longer period (Lie, 1973). Selective herbivory, predation, or parasitism generally suppresses the competitive capability of dominant species, allowing the coexistence of subordinate species and causing an increase in community diversity (Daleo, Alberti, Pascual, Canepuccia, & Iribarne, 2014; Hudson & Greenman, 1998; Iglesias et al., 2011; Ingram & Kirkpatrick, 2013; Kellogg & Bridgham, 2004; Lotze, Worm, & Sommer, 2000; Olff & Ritchie, 1998; Pierce, Luzzaro, Caccianiga, Ceriani, & Cerabolini, 2007; Roth, Whitford, & Steinberger, 2007; Santamaria, 2002; Smith et al., 2009). In contrast, nonselective herbivory, such as seed predation, may favor the dominant species and thus decrease overall diversity (Montgomery, 1980; Yu et al., 2014). Furthermore, differences in predation tolerance and resource requirements between dominant and subordinate species can affect the outcome of competition (Engelkes et al., 2016; Hendon & Briske, 2002; Kohyani, Bossuyt, Bonte, & Hoffmann, 2009; Lotze & Schramm, 2000).

The above mentioned selective foraging on dominant species is a common phenomenon in ecological systems. Why do the dominant tree taxa in zonal vegetation host more parasites than subordinate taxa do; that is, why do “the outstanding usually bear the brunt of attack?” One explanation is that dominants are generally apparent plants, which might attract more consumers (Dai et al., 2017). According to plant apparency, ecological apparency, and optimal foraging hypotheses, apparent dominants are more likely to be found and preferred by parasites, natural enemies, pollinators, and humans (Feeny, 1976; Gonçalves et al., 2016; Phillips & Gentry, 1993; Schlinkert et al., 2015). Plant dominance can facilitate the evolutionary adaptation of consumers, and many consumers use plant defensive compounds to locate host plants (Smilanich, Fincher, & Dyer, 2016).

The larvae of leafminers feed on and live inside leaf tissues between the upper and lower epidermis and produce distinct leaf mines, which may persist for many days (Hering, 1951; Liu, Dai, & Xu, 2015). Therefore, leaf mines might provide important insights regarding the life history, taxonomy, interspecific relationships, and evolution of leaf‐mining insects (Hirowatari, 2009; Liu et al., 2015). High incidences and abundances of leafminers on dominant plants have been demonstrated at global, regional, and community levels (Dai et al., 2017). For example, the highest reported abundance and richness values of leaf‐mining insects are found for members of Fagaceae and Myrtaceae (i.e., the most dominant plant families in the Northern and Southern Hemispheres, respectively) (Bairstow, Clarke, McGeoch, & Andrew, 2010; Claridge & Wilson, 1982; Dai, Xu, & Cai, 2014; Dai, Xu, & Ding, 2013; Faeth & Mopper, 1981; Ishida, Hattori, & Kimura, 2004; Kollár & Hrubík, 2009; Lopez‐Vaamonde, Godfray, & Cook, 2003; Nakamura, Hattori, Ishida, Sato, & Kimura, 2008; Opler & Davis, 1981; Sato, 1991; Sinclair & Hughes, 2008a,b). The variation in leafminer species richness among different host plants might be described by the species–area (i.e., leafminer species to host plant area) or species–apparency (i.e., leafminer species to host plant apparency) relationship (Dai et al., 2017; MacArthur & Wilson, 1967; Opler, 1974). “Area” here is a function of the distribution area, body size, number of individuals, and other indicators of plant dominance (Chaij, Devoto, Oleiro, Chaneton, & Mazía, 2016; Feeny, 1976; Joy & Crespi, 2012; Kamiya, O'Dwyer, Nakagawa, & Poulin, 2014; Miller, 2012). However, the unapparent relatives of apparent hosts might be utilized by leafminers due to the chemical similarities among phylogenetically closed plants (Dai et al., 2017). Therefore, the effects of plant phylogeny on the incidence of leafminers should be also considered (Claridge & Wilson, 1982; Dai et al., 2017; Godfray, 1984; Lawton & Price, 1979; Lopez‐Vaamonde et al., 2003).

In this study, we used Saihanwula National Nature Reserve as a case study to examine the presence–absence patterns of leaf‐mining insects on different plants in a forest‐grassland ecotone in Northeast China. To the best of our knowledge, there are fewer publications on the occurrence of leaf mining on different plants in East Asia than there are in Europe, America, and Australia. Different from our previous work on the relationship between plant apparency or phylogenetic isolation and plant utilization by leafminers and other consumers at the global scale (Dai et al., 2017), our hypothesis in this study is that dominant plant groups with high importance values are more susceptible to leaf‐mining insects at the regional level. Although there are many studies on leafminer species diversity based on plant characteristics, our study might be the first to use the importance value to study the leafminer species‐to‐area relationship. Moreover, in the previous work, we fit the dependence of consumer incidence on plant apparency or plant phylogeny separately (Dai et al., 2017), while in this study, we adopted phylogenetic generalized linear mixed model to consider plant apparency and plant phylogeny together in a model.

## 2 MATERIALS AND METHODS

### 2.1 Study area

The study was conducted in the Saihanwula National Nature Reserve, Inner Mongolia, China (43°59′–44°27′N, 118°18′–118°55′E). Its area is about 1000 km^2^. The climate is temperate semi‐arid, with long winters and short summers. The annual average temperature and rainfall are 2°C and 400 mm, respectively. The vegetation is in the transition zone between grassland and forest, and the forest types are transitional between the broad‐leaved forests of eastern Asia and the coniferous forests of the Greater Hinggan Mountains. Dominant trees include *Larix* spp., *Betula platyphylla*,* Quercus mongolica*,* Populus davidiana* and *Prunus sibirica*, and the dominant grasses are *Stipa baicalensis*,* Artemisia sacrorum*,* Filifolium sibiricum* and *Carex duriuscula* (Li, Zhang, & Bater, 2005; Li, Zhang, & Han, 1998; Zhang, 2007; Zheng, Gao, Teng, Feng, & Tian, 2015).

Saihanwula, as a National Natural Reserve, is under strict regulation and protection. Therefore, its vegetation has not changed as radically as the surrounding unprotected area. Moreover, host selection of leafminers might not only relate to the current general vegetation structure but may also show lags and accumulated responses to the plant composition of past decades (Godfray, 1984; Sugiura, 2010). It might be difficult to completely survey all vegetation again at the regional scale, as in the Saihanwula. In particular when considering only the presence–absence of leaf mine in a plant, the reuse of historical vegetation data might be reasonable at this stage.

### 2.2 Data collection

Plant attribute data, including importance value, were obtained from the records of Saihanwula Nature Reserve (Li et al., 1998, 2005; Zhang, 2007): (1) In each forest community type, a 20 × 20 m main plot was chosen. Trees were investigated individually within each 10 × 10 m subplot. Shrubs and tree seedlings were investigated in five subplots of 5 × 5 m at the four corners and the center of the main plot. Herbaceous species were investigated inside three 1 × 1 m subplots within each shrub subplot. (2) In each shrub community type, a 20 × 20 m main plot was chosen. Shrub or grass individuals were recorded within five 5 × 5 m subplots or three 1 × 1 m subplots, respectively, similar to the investigation conducted in the forest communities. (3) In each herbaceous community type, a 10 × 10 m main plot was chosen, twenty 1 × 1 m subplots were set up, and grass individuals were recorded.

The data were carefully reviewed and corrected for data consistency. The importance value (*IV*) of one tree species is the average of its relative density (*RD*), relative frequency (*RF*), and relative GBH (girth at breast height, i.e., 1.3 m from the ground; *RG*) (Equation (1)), whereas the *IV* of one grass species is the average of its *RD*,* RF,* and relative coverage (*RC*) (Equation (2)).

(1) $$IV=\left(RD+RF+RG\right)/3$$

(2) $$IV=\left(RD+RF+RC\right)/3$$

where *RD = * the density of a species/the total density of all species, *RF *= the frequency of a species/the sum of all frequencies, *RG *= the GBH of a tree species/the sum of all GBH values, and *RC *= the coverage of a grass species*/*the sum of all coverage values (Curtis & McIntosh, 1951; Gonmadje et al., 2011; Mori et al., 1983; Zhang, 2007).

We adopted the total importance value (*TIV*) to indicate the dominance or apparency of a plant species across all vegetation types in Saihanwula. The *TIV* of one plant species is the sum of all products of its *IV* in each community type and the area ratio (*AR*) of the corresponding community type (Equation (3)). The relative *TIV* (*RTIV*) of one plant species is the ratio of the *TIV* of one plant species to the *TIVs* of all plant species in Saihanwula (Equation (4)) (Li et al., 1998).

(3) $$TIV_{j}=\sum_{i=1}^{C}IV_{\mathrm{ij}}\times AR_{i}$$

(4) $$RTIV_{j}=\frac{TIV_{j}}{\sum_{j\;=\;1}^{S}TIV_{j}}\times 100\%$$

where *TIV*_*j*_ is the total importance value of plant species *j*,* RTIV*_*j*_ is the relative total importance value of plant species *j*,* IV*_*ij*_ is the importance value of species *j* in the *i*th community type, *AR*_*i*_ **= the area of the *i*th community type/the total area, *C* is the number of community types, and *S* is the number of plant species (Li et al., 1998).

The group importance value (*GIV*) of one plant group is the sum of the *TIVs* of all plant species in the group (Equation (5)). The relative *GIV* (*RGIV*) of one plant group is the ratio of the *TIVs* of all plant species in the group to the *TIVs* of all plant species in Saihanwula (Equation (6)) (Li et al., 1998). The group here could be categorized according to plant life form, water ecotype, phytogeographic distribution type, taxon (i.e., family or genus), and other plant attributes.

(5) $$GIV_{m}=\sum_{n=1}^{N_{m}}TIV_{n}$$

(6) $$RGIV_{m}=\frac{GIV_{m}}{\sum_{m=1}^{M}GIV_{m}}\times 100\%$$

where *GIV*_*m*_ is the total importance value of plant group *m*,* RTIV*_*m*_ is the relative total importance value of plant group *m*,* N*_*m*_ is the number of plant species in plant group *m*,* TIV*_*n*_ is the total importance value of plant species *n*, and *M* is the total number of plant groups (Li et al., 1998).

### 2.3 Host plant sampling

Leaf mines (i.e., the distinct feeding marks left by leafminers) can remain visible for a considerable period (Liu et al., 2015), including after larvae have emerged or after leaf fall. When we encountered damage on leaves from an inconclusive source, we carefully assessed whether the mesophyll tissues were eaten while both the upper and lower leaf epidermis were maintained (or at least the outer wall remaining undamaged) (Liu et al., 2015).

**Figure 1:**
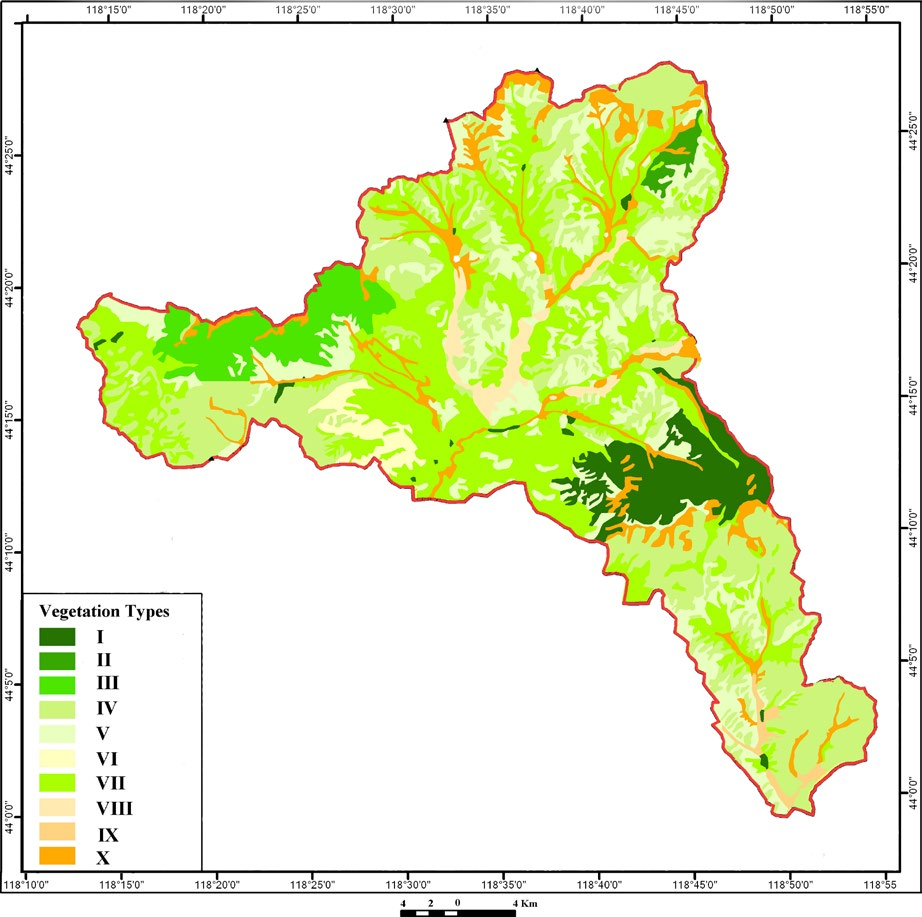
Vegetation map of Saihanwula National Nature Reserve. The map was modified from the original map produced by the Academy of Forestry Inventory and Planning of State Forestry Administration of China for Saihanwula National Nature Reserve Administration in 2013. Vegetation types are as follows: I. cold‐temperate deciduous needle‐leaved forest, II. cold‐temperate evergreen needle‐leaved forest, III. typical deciduous broad‐leaved forest, IV. montane *Populus*‐*Betula* deciduous forest, V. temperate deciduous broad‐leaved thicket, VI. montane evergreen broad‐leaved thicket, VII. meadow steppe, VIII. typical steppe, IX. forb meadow, and X. *Carex* meadow

Sampling sites and the corresponding survey trails were systematically chosen according to vegetation maps, historical data, and expert knowledge. Our sampling sites and trails covered and represented all 10 vegetation subtypes (cold‐temperate deciduous needle‐leaved forest, cold‐temperate evergreen needle‐leaved forest, typical deciduous broad‐leaved forest, montane *Populus*‐*Betula* deciduous forest, temperate deciduous broad‐leaved thicket, montane evergreen broad‐leaved thicket, meadow steppe, typical steppe, forb meadow, and *Carex* meadow) and most of the typical formations in the natural reserve (Figure 1). In July 2014 and October 2015, we (3–5 individuals per investigation group, with at least one experienced local guide) carefully examined all the trees, shrubs, and grasses that were visible along the studied trails and attempted to sample as many plant species with leaf mines as possible. Branches with mined leaves were collected and placed in plastic re‐sealable bags in the field. The host plants were then identified and recorded. Host plants and mined leaves were scanned, and their digital images were stored in our laboratory for future studies. When living larvae were found, we attempted to rear the mining species. During the studied period, if we could not find any leaf mines in one plant species, we assumed that leaf‐mining damage was absent from the plant species.

Our 8 years of experience with leafminer collection in China, which began in 2007, has made us thoroughly familiar with most types of leaf mines, allowing us to easily identify plants with leaf mines and some leafminer groups (Bai, Xu, & Dai, 2015, 2016; Dai et al., 2013, 2014, 2018; Liao, Liu, Xu, Staines, & Dai, 2018; Liu et al., 2015; Xu, Dai, Liao, Diškus, & Stonis, 2018; Xu et al., 2017). According to our rearing records and leaf mine characteristics, leafminers in Saihanwula belong to four insect orders: Lepidoptera (moths), Diptera (flies), Coleoptera (beetles), and Hymenoptera (sawflies). Dominant leafminer families and the corresponding genera are as follows: Nepticulidae: *Stigmella*; Agromyzidae: *Phytomyza*,* Agromyza*,* Liriomyza*; Gracillariidae: *Phyllonorycter*,* Caloptilia*,* Acrocercops*,* Cosmopterix*; Elachistidae: *Elachista*; Tischeriidae: *Tischeria*; Tenthredinidae: *Fenusa*,* Profenusa*; Curculionidae: *Rhynchaenus*; Buprestidae: *Trachys*; Coleophoridae: *Coleophora*; Heliozelidae; Lyonetiidae: *Lyonetia*; Psychidae; Bucculatricidae: *Bucculatrix*; Eriocraniidae; Gelechiidae; Yponomeutinae. Among these leafminers, some have only one generation per year, while others have two or more generations per year. Most leafminer species on deciduous trees or grasses finish their mining stages before late October and overwinter as pupae. In an interesting manner, some leaf‐mining larvae remain alive in the green islands on the dry or fallen leaves of some deciduous trees in Saihanwula. This provides the larvae with enough food to complete their life cycle before winter (Giron, Kaiser, Imbault, & Casas, 2007; Kaiser, Huguet, Casas, Commin, & Giron, 2010; Liu et al., 2015).

### 2.4 Data preparation

However, some leaf‐mining species and their life histories in China (including Saihanwula) remain unknown for the following reasons: (1) Many leaf mines were empty; (2) many leafminers died in transport or in the laboratory; (3) many leafminers were parasitized by parasitoid wasps; (4) some leafminer groups could not be identified at the species or even genus level as there were no available taxonomists with expertise in these groups, especially in the unfamiliar Chinese species; (5) no long‐term investigations of Chinese leafminers were officially performed on either the national or regional level beyond the preliminary work of our group. Moreover, there might be some types of gregarious leaf miners whose larvae share a single mine. Therefore, in this study, we had to consider the presence–absence of leaf mines at the regional level rather than the individual number, incidence rate, or leaf area damage. However, when we collect enough detailed data in the future, the latter quantitative parameters may provide more valuable information than the former binary presence–absence data, especially at the community level.

The presence or absence of leaf mines in each plant group was coded as binary data. Compared with abundance data, presence–absence data have several advantages: (1) Presence–absence data can increase efficiency in ecological and conservation research because they are easier to collect than abundance data and are much less costly in terms of time, price, and human resources, especially at large spatial or temporal scales (Badenhausser, Amouroux, & Bretagnolle, 2007; Casner, Forister, Ram, & Shapiro, 2014; Fukuda, Mouton, & De Baets, 2012; Furnas, 2013; Gu & Swihart, 2004; Gutiérrez, Harcourt, Díez, Gutiérrez Illán, & Wilson, 2013; Joseph, Field, Wilcox, & Possingham, 2006; MacKenzie & Nichols, 2004; Ribas & Padial, 2015). (2) In many cases, when differences among groups are large, presence–absence data can provide adequate indicators to describe ecological patterns, which are often in agreement with those obtained from abundance data (Carneiro, Bini, & Rodrigues, 2010; Landeiro et al., 2012; Melo, 2005; Ribas & Padial, 2015; Tweedley, Warwick, & Potter, 2015). (3) Presence–absence data can remove much of the noise induced by sampling biases or errors, whereas large sampling errors can lead to unreliable abundance data (Hirst & Jackson, 2007; Jackson & Harvey, 1997). (4) In some cases, only presence–absence data can be recorded, for example, when organisms grow clonally, are too abundant to count, or are difficult for nonexperts to identify taxonomically (Beisner, Peres‐Neto, Lindström, Barnett, & Longhi, 2006; Colwell, Chang, & Chang, 2004). (5) Presence–absence data are more appropriate than are abundance data for clarifying the effects of host characteristics on parasite similarity (Locke, Mclaughlin, & Marcogliese, 2013; Poulin, 2010; Poulin & Krasnov, 2010; Seifertová, Vyskočilová, Morand, & Šimková, 2008). We were not able to sample all of the leafminer species and their host plants within the short sampling period, but the use of presence–absence data may compensate for our sampling efforts. Moreover, these data might provide a rapid method to compare leaf‐mining patterns among different vegetation zones in China.

All plant species names, including host plant species names, were verified with the Taxonomic Name Resolution Service (TNRS), V 4.0 (Boyle et al., 2013). The plant names that could not be resolved at TNRS were verified at The Plant List (TPL), V 1.1 (http://www.theplantlist.org/).

### 2.5 Plant phylogeny and statistical analyses

As closely related organisms are more likely to share similar biological traits, PGLMMs (phylogenetic generalized linear mixed models) can be adopted to correct for phylogenetic effects (Gallien, Saladin, Boucher, Richardson, & Zimmermann, 2016; Ives & Garland, 2010; Paradis & Claude, 2002; Takemoto & Aie, 2017). To determine the relationship between the presence–absence of leaf mines on a given plant (as a binary variable) and the plant's *TIV* values, phylogenetic signal was measured, and phylogenetic logistic regression was performed. These procedures were performed using the binaryPGLMM function of the R package “rr2” and the phyloglm function of the R package “phylolm” (Ho & Ané, 2014; Ives & Garland, 2010, 2014; Ives, Helmus, & Ves, 2011; Paradis, Claude, & Strimmer, 2004). In the binaryPGLMM function, *s*2 is the scaling component of the variance in the PGLMM, where *s*2 = 0 suggests no phylogenetic signal and a high *s*2 value implies strong phylogenetic signal (Jamrozy et al., 2017). In the phyloglm function, *alpha* is the phylogenetic correlation parameter (an *alpha* value close to 0 suggests strong phylogenetic signal, *alpha *= 1 indicates a phylogenetic signal of trait evolution consistent with the expectation under Brownian motion, and an *alpha* value close to infinity implies low phylogenetic signal) (Blumstein et al., 2015; Gallien et al., 2016; O'Meara, Graham, Pellis, & Burghardt, 2015). Using the fitted coefficients from the phyloglm models, we plotted phylogenetic logistic regression curves using the plogis function of the R package “stats.” For comparison, we also fitted logistic link regressions of the presence–absence of leaf mines and *TIV* values using the glm function of the R package “stats.”

The plant phylogenetic trees, which were required for the above phylogenetic regression models, were constructed in the following way: The megatree R20120829mod.new and the corresponding ages of the main clades (Gastauer & Meira‐Neto, 2016) were updated to include all families of vascular plants. Therefore, lycophytes and their three extant families were added, and Athyriaceae was moved into the family Aspleniaceae (Christenhusz & Chase, 2014). The online Phylomatic program (http://phylodiversity.net/phylomatic/) was used to obtain the local plant phylogeny based on the megatree and our plant species list (Webb & Donoghue, 2005). Branch lengths were then adjusted with the Phylocom Bladj algorithm (Webb, Ackerly, & Kembel, 2008) based on the above modified ages file. A megatree of only plant families was also generated from the above modified megatree. Then, local plant phylogenies at the genus or family level were also obtained using the later megatree and our plant genus or family list. The R package “plantlist” (https://github.com/helixcn/plantlist/) was used to create a family/genus/species table for the Phylomatic software (Zhang, 2017). Note that the family/genus/genus or family/family/family table was generated for the plant genus or family list, respectively.

All of the plant species were ranked based on their *TIV* values (*TIV *> 0) and classified into 12 groups. Plant species with *TIV *= 0 were omitted from the following analyses. Then, the plant species were aggregated to the genus or family level. All of the plant genera/families were also ranked based on their *TIVs* and classified into a certain number of groups, but several plant genera/families with the smallest nonzero *TIVs* may have been omitted as the number of plant genera/families was not precisely divisible. We then counted the number of host plant species/genera/families in each ranked group and calculated the ratio of leafminer hosts.

The data analyses were mainly conducted in R 3.4.4 (R Core Team 2018) and RStudio 1.1.442 (RStudio Team 2018).

## 3 RESULTS

### 3.1 Roles of plant phylogeny and plant dominance

**Figure 2:**
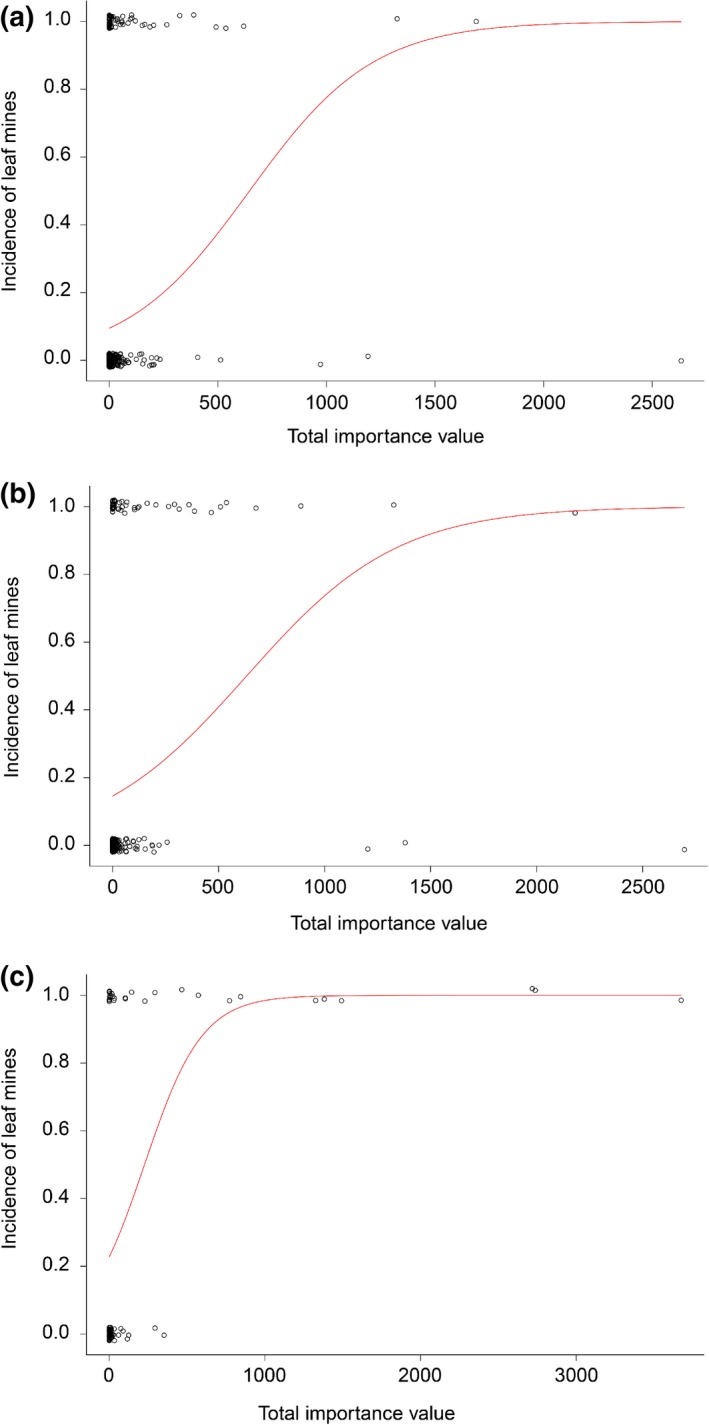
Phylogenetic generalized linear mixed models were fitted to show the incidence of leaf mines as a function of plant dominance (total importance value). (a) At the plant species level, (b) at the plant genus level, and (c) at the plant family level

Both phylogenetic logistic regression models exhibited a strong phylogenetic signal at the plant species, genus, and family level (binaryPGLMM: *s*2 = 0.320–0.453, *p *<* *0.001; phyloglm: *alpha *= 0.0021–0.0023; Table 1). After correcting for phylogenetic effects, a significant positive effect of *TIV* was observed on the presence–absence of leaf mines at the plant species, genus, and family level (binaryPGLMM: *B *=* *0.0018–0.0063, *p *<* *0.05; phyloglm: *B *= 0.0028–0.0054, *p *<* *0.05; Table 1). That is, the incidence probability of leaf mines among plant groups increased positively with *TIV* in a logistic way (Figure 2). Unexpectedly, the regression coefficients (*B*) of the nonphylogenetic logistic models (i.e., GLMs) were nearly equal to those obtained with binaryPGLMM, and the intercepts of the GLMs were nearly equal to those obtained with phyloglm (Table 1).

**Table 1:** binaryPGLMM, phyloglm, and GLM model statistics of the effect of total importance value on the presence–absence of leafminer on the plant group

Plant groups	binaryPGLMM						phyloglm						GLM				
	*B*	*p*(*B*)	Intercept	*p*(Intercept)	*s2*	*p*(*s2*)	*B*	*p*(*B*)	Intercept	*p*(Intercept)	*alpha*	AIC	*B*	*p*(*B*)	Intercept	*p*(Intercept)	AIC
species	0.0020	**0.003**	−3.83	**0.014**	0.453	**<0.001**	0.0035	**0.029**	−2.26	0.055	0.0021	561	0.0021	**0.003**	−2.26	**<0.001**	424
genus	0.0018	**0.007**	−3.10	**0.010**	0.439	**<0.001**	0.0028	**0.023**	−1.77	0.115	0.0021	370	0.0018	**0.005**	−1.77	**<0.001**	287
family	0.0063	**0.026**	−2.42	**0.009**	0.320	**<0.001**	0.0054	**0.008**	−1.22	0.164	0.0023	199	0.0063	**0.015**	−1.22	**<0.001**	90

Notes

*B* is the regression coefficient, *s2* is the scaling component of the variance in the PGLMM (phylogenetic generalized linear mixed model), *alpha* is the phylogenetic correlation parameter, and *AIC* is the Akaike Information Criterion score. Values in bold indicate a significant *p* value (*p *<* *0.05).

### 3.2 Relationship between plant importance value and host probability

Only those plant species/genera/families with nonzero importance values were considered. Dominant plant species tended to host leafminers (host ratio of 21.6%–45.9% for the top two ranked plant species groups with the highest *RGIVs*), whereas the remaining ranked plant species groups exhibited host ratios of 2.7%–16.2% (Table 2). Dominant plant genera also tended to host leafminers (host ratios of 33.3%–61.9% for the top three plant genus groups with the highest *RGIVs*), whereas the remaining ranked plant genus groups presented host ratios of 4.8%–23.8% (Table 3). Dominant plant families tended to host leafminers as well (host ratios of 100.0% for the first plant family group with the highest *RGIV*, i.e., all ten dominant plant families with the largest *GIVs* suffered leaf‐mining damage), whereas the remaining ranked plant family groups displayed host ratios of 10.0%–50.0% (Table 4; Figure 2c).

**Table 2:** Relationship between the total importance value of plant species groups and the ratio of leaf‐mining insect hosts among plant species

Rank of plant species group	Relative group importance value (*RGIV*)	Number of host plant species	Ratio of host plant species
1	81.82	17	0.459
2	9.66	8	0.216
3	4.55	3	0.081
4	2.20	3	0.081
5	1.19	6	0.162
6	0.41	4	0.108
7	0.10	4	0.108
8	0.04	3	0.081
9	0.02	4	0.108
10	0.01	1	0.027
11	0.01	2	0.054
12	0.00	3	0.081

A total of 444 plant species with available importance values were recorded in Saihanwula. These plant species were ranked based on their importance values and then classified into 12 groups (37 species per group). Host ratio* *= number of host species/total number of species in each group (i.e., 37).

**Table 3:** Relationship between the total importance value of plant genus groups and the ratio of leaf‐mining insect hosts among plant genera

Rank of plant genus group	Relative group importance value (*RGIV*)	Number of host plant genera	Ratio of host plant genera
1	80.29	13	0.619
2	11.61	7	0.333
3	4.51	7	0.333
4	1.82	1	0.048
5	1.01	3	0.143
6	0.56	5	0.238
7	0.13	3	0.143
8	0.03	3	0.143
9	0.02	2	0.095
10	0.01	1	0.048
11	0.00	2	0.095
12	0.00	3	0.143

A total of 254 plant genera with available importance values were recorded in Saihanwula. These plant genera were ranked based on their importance values and then classified into 12 groups (21 genera per group). The two plant genera with the smallest nonzero importance values were omitted. Host ratio* *= number of host genera/total number of genera in each group (i.e., 21).

**Table 4:** Relationship between the total importance value of plant family groups and the ratio of leaf‐mining insect hosts among plant families

Rank of plant family group	Relative group importance value (*RGIV*)	Number of host plant families	Ratio of host plant families
1	87.13	10	1.000
2	10.06	5	0.500
3	1.86	3	0.300
4	0.69	2	0.200
5	0.23	5	0.500
6	0.02	1	0.100
7	0.00	1	0.100

A total of 71 plant families with available importance values were recorded in Saihanwula. These plant families were ranked based on their importance values and then classified into seven groups (10 families per group). The plant family with the smallest nonzero importance value was omitted. Host ratio* *= number of host families/total number of families in each group (i.e., 10).

### 3.3 Impacts of plant life form, water ecotype, and phytogeographic distribution type

Trees were much more likely to be leafminer hosts (60.0%) than were shrubs, subshrubs, or grasses (<18.0%) (Table 5).

**Table 5:** Plant species of different life forms and information regarding their status as hosts of leaf‐mining insects

Life form	Relative group importance value (*RGIV*)	Total number of plant species	Number of host plant species	Host ratio
Perennials	48.34	458	32	0.070
Trees	28.48	25	15	0.600
Shrubs	13.57	51	9	0.176
Annuals and biennials	7.73	105	13	0.124
Subshrubs	1.88	12	0	0.000

Note

Host ratio* *= number of host species/total number of species.

Among the different water ecotypes, xeromesophytes, mesophytes, hygrophytes, mesoxerophytes, and hygromesophytes were more likely to exhibit leaf‐mining damage, while plants in extreme environments (hydrophytes and xerophytes) rarely hosted leafminers (Table 6).

**Table 6:** Plant species of different water ecotypes and information regarding their status as hosts of leaf‐mining insects

Water ecotype	Relative group importance value (*RGIV*)	Total number of plant species	Number of host plant species	Host ratio
Hydrophyte	0.00	2	0	0.000
Hygrophyte	0.72	35	4	0.114
Hygromesophyte	5.33	37	2	0.054
Mesophyte	74.01	369	47	0.127
Mesoxerophyte	10.86	81	8	0.099
Xeromesophyte	6.85	52	8	0.154
Xerophyte	2.23	75	2	0.027

Note

Host ratio* *= number of host species/total number of species.

Among the different phytogeographic distribution types, only those plant species belonging to the top seven types (East Palaearctic species, East Asia species, Palaearctic species, Holarctic species, Northeast China species, North China species, and Dahuric‐Mongolia species) with high *RGIVs* (>5.0) sustained damage from leaf‐mining insects (Table 7).

**Table 7:** Plant species of different phytogeographic distribution types and information regarding their status as hosts of leaf‐mining insects

Phytogeographic distribution type	Relative group importance value (*RGIV*)	Total number of plant species	Number of host plant species	Host ratio
East Palaearctic species	32.59	90	13	0.144
East Asia species	23.03	184	26	0.141
Palaearctic species	12.27	94	10	0.106
Holarctic species	8.84	95	9	0.095
Northeast China species	7.93	14	2	0.143
North China species	6.64	20	5	0.250
Dahuric‐Mongolia species	5.32	74	5	0.068
Eastern Siberia species	1.03	11	0	0.000
Cosmopolitan species	0.68	12	0	0.000
Unknown distribution type	0.66	11	0	0.000
Mongolia species	0.39	2	0	0.000
Europe‐Siberia species	0.30	5	0	0.000
Black Sea‐Kazakhstan‐Mongolia species	0.17	3	0	0.000
Kazakhstan‐Mongolia species	0.12	11	0	0.000
Central Asia species	0.04	16	0	0.000
East Asia‐North America species	0.00	2	0	0.000
Siberia species	0.00	1	0	0.000
Arctoalpine species	0.00	1	0	0.000
Tethys species	0.00	3	0	0.000
Tropicopolitan species	0.00	1	0	0.000
Yinshan‐Helan Mountain species	0.00	1	0	0.000

Note

Host ratio* *= number of host species/total number of species.

## 4 DISCUSSION

In this study, we measured plant dominance using the importance value, which is the sum of the relative density, relative frequency, and relative basal area of the plant group (Curtis & McIntosh, 1951). Relative density is related to the number of individuals, relative frequency is related to the distribution type, and the relative basal area is related to body size. As such, dominant plant groups with high importance values are abundant in number, exhibit a wide distribution, or are large in size. Previous studies have indicated that common plants are more likely to exhibit leaf‐mining damage than are rare plants, that widely distributed plants exhibit a higher leafminer incidence than do narrowly distributed plants, and that large plants with a complicated structure might be more vulnerable to leafminers than are small plants with a simple structure. For example, the number of leaf‐mining insects on Fagaceae plants in California is closely associated with the host distribution area (Opler, 1974). The distribution area and height of various tree species can partially explain differences in leafminer richness in Britain (Claridge & Wilson, 1982). A majority of the variation in species richness among agromyzid miners on Britain umbellifers was attributed to the distribution area, local abundance, number of habitats occupied, and body size of different host plants (Fowler, Lawton, Lawton, Fowler, & Lawton, 1982; Lawton & Price, 1979). Compared with normal *Q. falcata* saplings, smaller trees sprouting from root stalks near the ground hosted fewer miner species (Faeth & Simberloff, 1981; Lawton, 1983). At the global level, the presence of leaf‐mining chrysomelid beetles, tischeriid moths, agromyzid flies, and gracillariid moths strongly depends on the distribution range of plant families (Dai et al., 2017). In Saihanwula, the ratio of leafminer hosts among plants at the species, genus, and family level increased with the total importance value of the plant taxonomic group after phylogenetic correction. Thus, dominant plant taxonomic groups with high importance values were highly likely to host leafminers (Figure 2, Tables 1, 2, 3, 4). Among plant species of different phytogeographic distribution types in Saihanwula, widely distributed plant species showed high importance values and were likely to host leafminers, whereas narrowly distributed plant species exhibited the opposite patterns (Table 7). In general, dominant plant groups were more likely than their corresponding subordinate groups to suffer leaf‐mining damage. Our results are consistent with those of previous studies (Claridge & Wilson, 1982; Dai et al., 2017; Fowler et al., 1982; Lawton & Price, 1979; Opler, 1974).

Other factors may account for some variation in the species–area regression between plant dominance and leafminer incidence (Claridge & Wilson, 1982; Lawton & Price, 1979). In general, biotic factors play much important roles than abiotic ones in leaf‐mining distribution patterns (Sinclair & Hughes, 2008a). Plant phylogeny, which is highly related to plant chemistry, may have large influences on the species–area relationship of leafminers (Claridge & Wilson, 1982; Godfray, 1984). Among the plant species of different life forms, tree groups did not exhibit the highest total importance values but were much more likely to suffer leaf‐mining damage than any other life form in Saihanwula (Table 5). Among plant species of different water ecotypes in Saihanwula, plants in extremely dry or wet environments had very little likelihood of hosting leafminers (Table 6). In the same way, no leafminers were discovered at two driest places in Australia (Sinclair & Hughes, 2008a); aquatic habitats may be unfavorable for the agromyzid leafminers (Lawton & Price, 1979). The presence–absence of leaf mining might be obviously related to leaf physical traits such as leaf size, leaf length, leaf thickness, or leaf form (Dai, Zhu, Xu, Liu, & Wang, 2011; Fowler et al., 1982; Godfray, 1984; Lawton & Price, 1979; Sinclair & Hughes, 2008a). Adult leafminers should lay eggs on leaves that are large enough for the larvae to complete their life histories (Dai et al., 2011). Therefore, many leafminers prefer larger leaves to smaller ones (Faeth, 1991; Hileman & Lieto, 1981). In contrast, plant phylogenetic isolation, life history, interspecific competition, and natural enemies had no important impacts on the number of agromyzid flies on the British Umbelliferae (Lawton & Price, 1979).

Although the influence of importance value on the presence–absence of leaf mines was not independent of plant phylogenetic relationships, the role of plant dominance on the probability of being mined was clear (Table 1, Figure 2). One possible explanation for the similar regression coefficients or intercepts between the PGLMMs and nonphylogenetic logistic models is that the close relatives of the dominant plants were more dominant than the other plants and were thus more susceptible to plant parasites.

Vegetation parameters such as density, frequency, coverage, diversity, and importance value have been used to measure the apparency or dominance of plant species (Gonçalves et al., 2016; Guo & Rundel, 1997). Higher dominance is associated with more host–consumer encounters (random placement hypothesis) and more ecological niches for consumers (habitat diversity hypothesis) (Miller, 2012; Strona & Fattorini, 2014). For example, the occurrence of more species in a plant family implies the existence of a greater number of available niches (de Araújo, dos Santos, & Gomes‐Klein, 2012; de Araújo, Silva, dos Santos, & Gomes‐klein, 2013; Joy & Crespi, 2012; Mendonça, 2007). Therefore, according to the plant family size hypothesis, larger plant families are expected to host more parasites (de Araújo, 2011; de Araújo et al., 2012, 2013; Cuevas‐Reyes, Quesada, Hanson, & Oyama, 2007; Dai et al., 2017; Fernandes, 1992; Gonçalves‐Alvim, Fernandes, & Goncalves‐Alvim, 2001; Lawton & Price, 1979; Mendonça, 2007; Price, 1977; Veldtman & McGeoch, 2003; Ward & Spalding, 1993). In general, high dominance can be related to a high risk of pest or pathogen attack. As the importance value encompasses several plant traits related to plant dominance, it is expected to be a valid predictor of consumer occurrence (de Albuquerque & de Lucena, 2005; de Lucena, de Lima Araújo, & de Albuquerque, 2007; de Lucena, de Medeiros, Araújo, Alves, & de Albuquerque, 2012), as verified in the present study.

In summary, dominant plant groups are large and susceptible targets for leaf‐mining insects even when we consider the effects of plant phylogeny and other plant attributes. Such a consistent leaf‐mining distribution pattern across different countries, vegetation types and plant taxa can be explained by the “species‐area relationship” (i.e., the leafminer species incidence to plant importance value relationship) or the “species‐apparency relationship.”

## DATA ACCESSIBILITY

Data for this study are available from the Dryad Digital Repository: https://doi.org/10.5061/dryad.sc3fr20

## CONFLICT OF INTEREST

None declared.

## AUTHOR CONTRIBUTIONS

X.D. conceived and designed the study, performed the fieldwork, managed the project, analyzed the data, and wrote the manuscript. C.L. extracted the data from publications, rechecked the field data, reanalyzed all the data, and modified the vegetation map. J.X. helped identify leaf mines and guide the writing of Chinese version manuscript. Q.G. helped to analyze the data and improve the manuscript in English. W.Z. performed the fieldwork and analyzed the data. Z.Z. wrote the first version of the manuscript in Chinese. Bater provided the background data of Saihanwula and aided the fieldwork.

## References

[ece34284-bib-0001] Badenhausser, I. , Amouroux, P. , & Bretagnolle, V. (2007). Estimating acridid densities in grassland habitats: A comparison between presence‐absence and abundance sampling designs. Environmental Entomology, 36, 1494–1503. 10.1603/0046-225X(2007)36[1494:EADIGH]2.0.CO;2 18284778

[ece34284-bib-0002] Bai, H. , Xu, J. , & Dai, X. (2015). Three new species, two newly recorded species and one newly recorded genus of Lithocolletinae (Lepidoptera: Gracillariidae) from China. Zootaxa, 4032, 229–235. 10.11646/zootaxa.4032.2.10 26624356

[ece34284-bib-0003] Bai, H. , Xu, J. , & Dai, X. (2016). Two new and one newly recorded species of Gracillariidae from China (Lepidoptera). ZooKeys, 559, 139–150. 10.3897/zookeys.559.6812 PMC4768276 27006609

[ece34284-bib-0004] Bairstow, K. A. , Clarke, K. L. , McGeoch, M. A. , & Andrew, N. R. (2010). Leaf miner and plant galler species richness on Acacia: Relative importance of plant traits and climate. Oecologia, 163, 437–448. 10.1007/s00442-010-1606-4 20349248

[ece34284-bib-0005] Behera, S. K. , Sahu, N. , Mishra, A. K. , Bargali, S. S. , Behera, M. D. , & Tuli, R. (2017). Aboveground biomass and carbon stock assessment in Indian tropical deciduous forest and relationship with stand structural attributes. Ecological Engineering, 99, 513–524. 10.1016/j.ecoleng.2016.11.046

[ece34284-bib-0006] Beisner, B. E. , Peres‐Neto, P. R. , Lindström, E. S. , Barnett, A. , & Longhi, M. L. (2006). The role of environmental and spatial processes in structuring lake communities from bacteria to fish. Ecology, 87, 2985–2991. 10.1890/0012-9658(2006)87[2985:TROEAS]2.0.CO;2 17249222

[ece34284-bib-0007] Blumstein, D. T. , Buckner, J. , Shah, S. , Patel, S. , Alfaro, M. E. , & Natterson‐Horowitz, B. (2015). The evolution of capture myopathy in hooved mammals: A model for human stress cardiomyopathy? Evolution, Medicine, and Public Health, 2015, 195–203. 10.1093/emph/eov015 PMC4538952 26198189

[ece34284-bib-0008] Bouchenak‐Khelladi, Y. , Slingsby, J. A. , Verboom, G. A. , & Bond, W. J. (2014). Diversification of C4 grasses (Poaceae) does not coincide with their ecological dominance. American Journal of Botany, 101, 300–307. 10.3732/ajb.1300439 24509796

[ece34284-bib-0009] Boyle, B. , Hopkins, N. , Lu, Z. , Raygoza Garay, J. A. , Mozzherin, D. , Rees, T. , … Weakley, A. (2013). The taxonomic name resolution service: An online tool for automated standardization of plant names. BMC Bioinformatics, 14, 16 10.1186/1471-2105-14-16 23324024 PMC3554605

[ece34284-bib-0010] Brandt, R. , Zimmermann, H. , Hensen, I. , Mariscal Castro, J. C. , & Rist, S. (2012). Agroforestry species of the Bolivian Andes: An integrated assessment of ecological, economic and socio‐cultural plant values. Agroforestry Systems, 86, 1–16. 10.1007/s10457-012-9503-y

[ece34284-bib-0011] Carneiro, F. M. , Bini, L. M. , & Rodrigues, L. C. (2010). Influence of taxonomic and numerical resolution on the analysis of temporal changes in phytoplankton communities. Ecological Indicators, 10, 249–255. 10.1016/j.ecolind.2009.05.004

[ece34284-bib-0012] Casner, K. L. , Forister, M. L. , Ram, K. , & Shapiro, A. M. (2014). The utility of repeated presence data as a surrogate for counts: A case study using butterflies. Journal of Insect Conservation, 18, 13–27. 10.1007/s10841-013-9610-8

[ece34284-bib-0013] Chaij, J. , Devoto, M. , Oleiro, M. , Chaneton, E. J. , & Mazía, N. (2016). Complexity of leaf miner‐parasitoid food webs declines with canopy height in Patagonian beech forests. Ecological Entomology, 41, 599–610. 10.1111/een.12332

[ece34284-bib-0014] Chalmandrier, L. , Münkemüller, T. , Lavergne, S. , & Thuiller, W. (2015). Effects of species ‘similarity and dominance on the functional and phylogenetic structure of a plant meta‐community. Ecology, 96, 143–153. 10.1890/13-2153.1 26236899 PMC4539579

[ece34284-bib-0015] Christenhusz, M. J. M. , & Chase, M. W. (2014). Trends and concepts in fern classification. Annals of Botany, 113, 571–594. 10.1093/aob/mct299 24532607 PMC3936591

[ece34284-bib-0016] Christianou, M. , & Ebenman, B. (2005). Keystone species and vulnerable species in ecological communities: Strong or weak interactors? Journal of Theoretical Biology, 235, 95–103. 10.1016/j.jtbi.2004.12.022 15833316

[ece34284-bib-0017] Claridge, M. F. , & Wilson, M. R. (1982). Insect herbivore guilds and species‐area relationships: Leafminers on British trees. Ecological Entomology, 7, 19–30. 10.1111/j.1365-2311.1982.tb00640.x

[ece34284-bib-0018] Collins, O. C. , & Duffy, K. J. (2016). Consumption threshold used to investigate stability and ecological dominance in consumer‐resource dynamics. Ecological Modelling, 319, 155–162. 10.1016/j.ecolmodel.2015.03.021

[ece34284-bib-0019] Colwell, R. K. , Chang, X. M. , & Chang, J. (2004). Interpolating, extrapolating, and comparing incidence‐based species accumulation curves. Ecology, 85, 2717–2727. 10.1890/03-0557

[ece34284-bib-0020] Cuevas‐Reyes, P. , Quesada, M. , Hanson, P. , & Oyama, K. (2007). Interactions among three trophic levels and diversity of parasitoids: A case of top‐down processes in Mexican tropical dry forest. Environmental Entomology, 36, 792–800. 10.1093/ee/36.4.792 17716469

[ece34284-bib-0021] Curtis, J. T. , & McIntosh, R. P. (1951). An upland forest continuum in the prairie‐forest border region of Wisconsin. Ecology, 32, 476–496. 10.2307/1931725

[ece34284-bib-0022] Dahdouh‐Guebas, F. , Koedam, N. , Satyanarayana, B. , & Cannicci, S. (2011). Human hydrographical changes interact with propagule predation behaviour in Sri Lankan mangrove forests. Journal of Experimental Marine Biology and Ecology, 399, 188–200. 10.1016/j.jembe.2010.11.012

[ece34284-bib-0023] Dahdouh‐Guebas, F. , Verheyden, A. , De Genst, W. , Hettiarachchi, S. , & Koedam, N. (2000). Four decade vegetation dynamics in Sri Lankan mangrove as detected from sequential aerial photography: A case study in Galle. Bulletin of Marine Science, 67, 741–759.

[ece34284-bib-0024] Dai, X. , Xu, J. , & Cai, L. (2014). Effects of roads on *Castanopsis carlesii* seedlings and their leaf herbivory in a subtropical forest in China. Journal of Insect Science, 14, 17. 25373164 10.1093/jis/14.1.17 PMC4199530

[ece34284-bib-0025] Dai, X. , Xu, J. , & Ding, X. (2013). Circular distribution pattern of plant modulars and endophagous herbivory within tree crowns: The impact of roadside light conditions. Journal of Insect Science, 13, 141. 24794427 10.1673/031.013.14101 PMC4015414

[ece34284-bib-0026] Dai, X. , Xu, J. , Guo, Q. , Lai, S. , Liu, P. , Fan, J. , & Tang, P. (2018). Density effect and intraspecific competition in a leaf‐mining moth on bamboo leaves. Journal of Forestry Research, 29 online first. 10.1007/s11676-018-0655-0

[ece34284-bib-0027] Dai, X. , Zhang, W. , Xu, J. , Duffy, K. J. , & Guo, Q. (2017). Global pattern of plant utilization across different organisms: Does plant apparency or plant phylogeny matter? Ecology and Evolution, 7, 2535–2545. 10.1002/ece3.2882 28428845 PMC5395452

[ece34284-bib-0028] Dai, X. , Zhu, C. , Xu, J. , Liu, R. , & Wang, X. (2011). Effects of physical leaf features of host plants on leaf‐mining insects. Shengtai Xuebao/Acta Ecologica Sinica, 31, 1440–1449.

[ece34284-bib-0029] Daleo, P. , Alberti, J. , Pascual, J. , Canepuccia, A. , & Iribarne, O. (2014). Herbivory affects salt marsh succession dynamics by suppressing the recovery of dominant species. Oecologia, 175, 335–343. 10.1007/s00442-014-2903-0 24549938

[ece34284-bib-0030] de Albuquerque, U. P. , & de Lucena, R. F. P. (2005). Can apparency affect the use of plants by local people in tropical forests? Interciencia, 30, 506–510.

[ece34284-bib-0031] de Araújo, W. S. (2011). Size, age and composition: Characteristics of plant taxa as diversity predictors of gall‐midges (Diptera: Cecidomyiidae). Revista de Biologia Tropical, 59, 1599–1607. 22208077 10.15517/rbt.v59i4.3423

[ece34284-bib-0032] de Araújo, W. S. , dos Santos, B. B. , & Gomes‐Klein, V. L. (2012). Relationship between host plant diversity and gall‐inducing insect's richness in the Brazilian Cerrado. Neotropical Biology and Conservation, 7, 41–47.

[ece34284-bib-0033] de Araújo, W. S. , Silva, I. P. A. , dos Santos, B. B. , & Gomes‐klein, V. L. (2013). Host plants of insect‐induced galls in areas of cerrado in the state of Goiás, Brazil. Acta Botanica Brasilica, 27, 537–542. 10.1590/S0102-33062013000300011

[ece34284-bib-0034] de Lucena, R. F. P. , de Lima Araújo, E. , & de Albuquerque, U. P. (2007). Does the local availability of woody Caatinga plants (Northeastern Brazil) explain their use value? Economic Botany, 61, 347–361. 10.1663/0013-0001(2007)61[347:DTLAOW]2.0.CO;2

[ece34284-bib-0035] de Lucena, R. F. P. , de Medeiros, P. M. , Araújo, E. L. , Alves, A. G. C. , & de Albuquerque, U. P. (2012). The ecological apparency hypothesis and the importance of useful plants in rural communities from Northeastern Brazil: An assessment based on use value. Journal of Environmental Management, 96, 106–115. 10.1016/j.jenvman.2011.09.001 22208403

[ece34284-bib-0036] Endress, B. A. , Naylor, B. J. , Parks, C. G. , & Radosevich, S. R. (2007). Landscape factors influencing the abundance and dominance of the invasive plant *Potentilla Recta* . Rangeland Ecology & Management, 60, 218–224. 10.2111/1551-5028(2007)60[218:LFITAA]2.0.CO;2

[ece34284-bib-0037] Engelkes, T. , Meisner, A. , Morriën, E. , Kostenko, O. , Van der Putten, W. H. , & Macel, M. (2016). Herbivory and dominance shifts among exotic and congeneric native plant species during plant community establishment. Oecologia, 180, 507–517. 10.1007/s00442-015-3472-6 26481795 PMC4723625

[ece34284-bib-0038] Engemann, K. , Sandel, B. , Enquist, B. J. , Jørgensen, P. M. , Kraft, N. , Marcuse‐Kubitza, A. , … Svenning, J. C. (2016). Patterns and drivers of plant functional group dominance across the Western Hemisphere: A macroecological re‐assessment based on a massive botanical dataset. Botanical Journal of the Linnean Society, 180, 141–160. 10.1111/boj.12362

[ece34284-bib-0039] Faeth, S. H. (1991). Effect of oak leaf size on abundance, dispersion, and survival of the leafminer Cameraria sp. (Lepidoptera: Gracillariidae). Environmental Entomology, 20, 196–204. 10.1093/ee/20.1.196

[ece34284-bib-0040] Faeth, S. , & Mopper, S. (1981). Abundances and diversity of leaf‐mining insects on three oak host species: Effects of host‐plant phenology and nitrogen content of leaves. Oikos, 37, 238–251. 10.2307/3544471

[ece34284-bib-0041] Faeth, S. H. , & Simberloff, D. (1981). Experimental isolation of oak host plants: Effects on mortality, survivorship, and abundances of leaf‐mining insects. Ecology, 62, 625–635. 10.2307/1937730

[ece34284-bib-0042] Feeny, P. (1976). Plant apparency and chemical defense. Recent Advances in Phytochemistry, 10, 1–40.

[ece34284-bib-0043] Fernandes, G. W. (1992). Plant family size and age effects on insular gall‐forming species richness. Global Ecology and Biogeography Letters, 2, 71–74. 10.2307/2997508

[ece34284-bib-0044] Fowler, S. V. , Lawton, J. H. , Lawton, F. , Fowler, S. V. , & Lawton, J. H. (1982). The effects of host‐plant distribution and local abundance on the species richness of agromyzid flies attacking British umbellifers. Ecological Entomology, 7, 257–265. 10.1111/j.1365-2311.1982.tb00665.x

[ece34284-bib-0045] Frieswyk, C. B. , Johnston, C. A. , & Zedler, J. B. (2007). Identifying and characterizing dominant plants as an indicator of community condition. Journal of Great Lakes Research, 33, 125–135. 10.3394/0380-1330(2007)33[125:IACDPA]2.0.CO;2

[ece34284-bib-0046] Fukuda, S. , Mouton, A. M. , & De Baets, B. (2012). Abundance versus presence/absence data for modelling fish habitat preference with a genetic Takagi‐Sugeno fuzzy system. Environmental Monitoring and Assessment, 184, 6159–6171. 10.1007/s10661-011-2410-2 22068315

[ece34284-bib-0047] Furey, C. , Tecco, P. A. , Perez‐Harguindeguy, N. , Giorgis, M. A. , & Grossi, M. (2014). The importance of native and exotic plant identity and dominance on decomposition patterns in mountain woodlands of central Argentina. Acta Oecologica, 54, 13–20. 10.1016/j.actao.2012.12.005

[ece34284-bib-0048] Furnas, B. J. (2013). Survey and analytical methods for long‐term monitoring of wildlife metacommunities in California Montane forests. Doctoral dissertation. Berkeley, CA: University of California, Berkeley.

[ece34284-bib-0049] Gallien, L. , Saladin, B. , Boucher, F. C. , Richardson, D. M. , & Zimmermann, N. E. (2016). Does the legacy of historical biogeography shape current invasiveness in pines? New Phytologist, 209, 1096–1105. 10.1111/nph.13700 26477339

[ece34284-bib-0050] Gastauer, M. , & Meira‐Neto, J. A. A. (2016). An enhanced calibration of a recently released megatree for the analysis of phylogenetic diversity. Brazilian Journal of Biology, 76, 619–628. 10.1590/1519-6984.20814 27097100

[ece34284-bib-0051] Giron, D. , Kaiser, W. , Imbault, N. , & Casas, J. (2007). Cytokinin‐mediated leaf manipulation by a leafminer caterpillar. Biology letters, 3, 340–343. 10.1098/rsbl.2007.0051 17412674 PMC2390681

[ece34284-bib-0052] Godfray, H. C. J. (1984). Patterns in the distribution of leaf‐miners on British trees. Ecological Entomology, 9, 163–168. 10.1111/j.1365-2311.1984.tb00711.x

[ece34284-bib-0053] Gonçalves, P. H. S. , Albuquerque, U. P. , & de Medeiros, P. M. (2016). The most commonly available woody plant species are the most useful for human populations: A meta‐analysis. Ecological Applications, 26, 2238–2253. 10.1002/eap.1364 27755717

[ece34284-bib-0054] Gonçalves‐Alvim, S. J. , Fernandes, G. W. , & Goncalves‐Alvim, S. J. (2001). Biodiversity of galling insects: Historical, community and habitat effects in four neotropical savannas. Biodiversity and Conservation, 10, 79–98. 10.1023/A:1016602213305

[ece34284-bib-0055] Gonmadje, C. F. , Doumenge, C. , McKey, D. , Tchouto, G. P. M. , Sunderland, T. C. H. , Balinga, M. P. B. , & Sonké, B. (2011). Tree diversity and conservation value of Ngovayang's lowland forests, Cameroon. Biodiversity and Conservation, 20, 2627–2648. 10.1007/s10531-011-0095-z

[ece34284-bib-0056] Greig‐Smith, P. (1983). Quantitative plant ecology. Berkeley, CA: University of California Press.

[ece34284-bib-0057] Grime, J. P. (1998). Benefits of plant diversity to ecosystems: Immediate, filter and founder effects. Journal of Ecology, 86, 902–910. 10.1046/j.1365-2745.1998.00306.x

[ece34284-bib-0058] Gu, W. , & Swihart, R. K. (2004). Absent or undetected? Effects of non‐detection of species occurrence on wildlife‐habitat models. Biological Conservation, 116, 195–203. 10.1016/S0006-3207(03)00190-3

[ece34284-bib-0059] Guèze, M. , Luz, A. C. , Paneque‐Gálvez, J. , Macía, M. J. , Orta‐Martínez, M. , Pino, J. , & Reyes‐García, V. (2014). Are ecologically important tree species the most useful? A case study from indigenous people in the Bolivian Amazon. Economic Botany, 68, 1–15. 10.1007/s12231-014-9257-8 26097243 PMC4471144

[ece34284-bib-0060] Guo, Z. G. , Li, X. F. , Liu, X. Y. , & Zhou, X. R. (2012). Response of alpine meadow communities to burrow density changes of plateau pika (Ochotona curzoniae) in the Qinghai‐Tibet Plateau. Acta Ecologica Sinica, 32, 44–49. 10.1016/j.chnaes.2011.12.002

[ece34284-bib-0061] Guo, Q. , & Rundel, P. W. (1997). Measuring dominance and diversity in ecological communities: Choosing the right variables. Journal of Vegetation Science, 8, 405–408. 10.2307/3237331

[ece34284-bib-0062] Gutiérrez, D. , Harcourt, J. , Díez, S. B. , Gutiérrez Illán, J. , & Wilson, R. J. (2013). Models of presence‐absence estimate abundance as well as (or even better than) models of abundance: The case of the butterfly Parnassius apollo. Landscape Ecology, 28, 401–413. 10.1007/s10980-013-9847-3

[ece34284-bib-0063] Hendon, B. C. , & Briske, D. D. (2002). Relative herbivory tolerance and competitive ability in two dominant: Subordinate pairs of perennial grasses in a native grassland. Plant Ecology, 160, 43–51. 10.1023/A:1015841214866

[ece34284-bib-0064] Henkel, T. K. , Chambers, J. Q. , & Baker, D. A. (2016). Delayed tree mortality and Chinese tallow (*Triadica sebifera*) population explosion in a Louisiana bottomland hardwood forest following Hurricane Katrina. Forest Ecology and Management, 378, 222–232. 10.1016/j.foreco.2016.07.036

[ece34284-bib-0065] Hering, E. M. (1951). Biology of the Leaf Miners. Berlin, Germany: Dr. W. Junk Gravenhage 10.1007/978-94-015-7196-8

[ece34284-bib-0066] Hileman, D. R. , & Lieto, L. F. (1981). Mortality and area reduction in leaves of the bog shrub Chamaedaphne calyculata (Ericaceae) caused by the leaf miner Coptodisca kalmiella (Lepidoptera: Heliozelidae). American Midland Naturalist, 106, 180–188. 10.2307/2425147

[ece34284-bib-0067] Hirowatari, T. (2009). Biology of leaf mining insects. Nature & Insects, 44, 2–3.

[ece34284-bib-0068] Hirst, C. N. , & Jackson, D. A. (2007). Reconstructing community relationships: The impact of sampling error, ordination approach, and gradient length. Diversity and Distributions, 13, 361–371. 10.1111/j.1472-4642.2007.00307.x

[ece34284-bib-0069] Ho, L. S. T. , & Ané, C. (2014). A linear‐time algorithm for gaussian and non‐gaussian trait evolution models. Systematic Biology, 63, 397–408. 24500037 10.1093/sysbio/syu005

[ece34284-bib-0070] Hu, Y. , Su, Z. , Li, W. , Li, J. , & Ke, X. (2015). Influence of tree species composition and community structure on carbon density in a subtropical forest. PLoS ONE, 10, e0136984 10.1371/journal.pone.0136984 26317523 PMC4552639

[ece34284-bib-0071] Hudson, P. , & Greenman, J. (1998). Competition mediated by parasites: Biological and theoretical progress. Trends in Ecology and Evolution, 13, 387–390. 10.1016/S0169-5347(98)01475-X 21238357

[ece34284-bib-0072] Hurlbert, S. H. (1997). Functional importance vs keystoneness: Reformulating some questions in theoretical biocenology. Austral Ecology, 22, 369–382. 10.1111/j.1442-9993.1997.tb00687.x

[ece34284-bib-0073] Iglesias, C. , Mazzeo, N. , Meerhoff, M. , Lacerot, G. , Clemente, J. M. , Scasso, F. , … Jeppesen, E. (2011). High predation is of key importance for dominance of small‐bodied zooplankton in warm shallow lakes: Evidence from lakes, fish exclosures and surface sediments. Hydrobiologia, 667, 133–147. 10.1007/s10750-011-0645-0

[ece34284-bib-0074] Ingram, J. , & Kirkpatrick, J. B. (2013). Native vertebrate herbivores facilitate native plant dominance in old fields while preventing native tree invasion ‐ Implications for threatened species. Pacific Conservation Biology, 19, 331–342. 10.1071/PC130331

[ece34284-bib-0075] Ishida, T. A. , Hattori, K. , & Kimura, M. T. (2004). Abundance of leafminers and leaf area loss by chewing herbivores in hybrids between *Quercus crispula* and *Quercus dentata* . Canadian Journal of Forest Research, 34, 2501–2507. 10.1139/x04-132

[ece34284-bib-0076] Ives, A. R. , & Garland, T. (2010). Phylogenetic logistic regression for binary dependent variables. Systematic Biology, 59, 9–26. 10.1093/sysbio/syp074 20525617

[ece34284-bib-0077] Ives, A. R. , & Garland, T. (2014). Phylogenetic regression for binary dependent variables In Garamszegi L. Z. (Ed.), Modern phylogenetic comparative methods and their application in evolutionary biology: Concepts and practice (pp. 231–261). Berlin, Heidelberg: Springer Berlin Heidelberg.

[ece34284-bib-0078] Ives, A. R. , Helmus, M. R. , & Ves, A. N. R. I. (2011). Generalized linear mixed models for phylogenetic analyses of community structure. Ecological Monographs, 81, 511–525. 10.1890/10-1264.1

[ece34284-bib-0079] Jackson, D. A , & Harvey, H. H. (1997). Qualitative and quantitative sampling of lake fish communities. Canadian Journal of Fisheries and Aquatic Sciences, 54, 2807–2813. 10.1139/f97-182

[ece34284-bib-0080] Jamrozy, D. , Coll, F. , Mather, A. E. , Harris, S. R. , Harrison, E. M. , MacGowan, A. , … Peacock, S. J. (2017). Evolution of mobile genetic element composition in an epidemic methicillin‐resistant *Staphylococcus aureus*: Temporal changes correlated with frequent loss and gain events. BMC Genomics, 18, 684 10.1186/s12864-017-4065-z 28870171 PMC5584012

[ece34284-bib-0081] Joseph, L. N. , Field, S. A. , Wilcox, C. , & Possingham, H. P. (2006). Presence‐absence versus abundance data for monitoring threatened species. Conservation Biology, 20, 1679–1687. 10.1111/j.1523-1739.2006.00529.x 17181803

[ece34284-bib-0082] Joy, J. B. , & Crespi, B. J. (2012). Island phytophagy: Explaining the remarkable diversity of plant‐feeding insects. Proceedings of the Royal Society B: Biological Sciences, 279, 3250–3255. 10.1098/rspb.2012.0397 22553094 PMC3385722

[ece34284-bib-0083] Kaiser, W. , Huguet, E. , Casas, J. , Commin, C. , & Giron, D. (2010). Plant green‐island phenotype induced by leaf‐miners is mediated by bacterial symbionts. Proceedings. Biological sciences/The Royal Society, 277, 2311–2319. 10.1098/rspb.2010.0214 PMC2894905 20356892

[ece34284-bib-0084] Kamiya, T. , O'Dwyer, K. , Nakagawa, S. , & Poulin, R. (2014). What determines species richness of parasitic organisms? A meta‐analysis across animal, plant and fungal hosts. Biological Reviews, 89, 123–134. 10.1111/brv.12046 23782597

[ece34284-bib-0085] Kellogg, C. H. , & Bridgham, S. D. (2004). Disturbance, herbivory, and propagule dispersal control dominance of an invasive grass. Biological Invasions, 6, 319–329. 10.1023/B:BINV.0000034606.84830.d5

[ece34284-bib-0086] Kent, M. (2012). Vegetation description and data analysis: A practical approach, 2nd Edn.. Oxford, UK: John Wiley & Sons Ltd.

[ece34284-bib-0087] Khairil, M. , Juliana, W. A. W. , Nizam, M. S. , Wan Juliana, W. A. , & Nizam, M. S. (2014). Edaphic influences on tree species composition and community structure in a tropical watershed forest in peninsular Malaysia. Journal of Tropical Forest Science, 26, 284–294.

[ece34284-bib-0088] Kohyani, P. T. , Bossuyt, B. , Bonte, D. , & Hoffmann, M. (2009). Differential herbivory tolerance of dominant and subordinate plant species along gradients of nutrient availability and competition (ed AG Van der Valk). Plant Ecology, 201, 611–619. 10.1007/s11258-008-9515-x

[ece34284-bib-0089] Koike, F. (2001). Plant traits as predictors of woody species dominance in climax forest communities. Journal of Vegetation Science, 12, 327–336. 10.2307/3236846

[ece34284-bib-0090] Kollár, J. , & Hrubík, P. (2009). The mining species on woody plants of urban environments in the West Slovak area. Acta Entomologica Serbica, 14, 83–91.

[ece34284-bib-0091] Kunte, K. (2008). Competition and species diversity: Removal of dominant species increases diversity in Costa Rican butterfly communities. Oikos, 117, 69–76. 10.1111/j.2007.0030-1299.16125.x

[ece34284-bib-0092] Landeiro, V. L. , Bini, L. M. , Costa, F. R. C. , Franklin, E. , Nogueira, A. , De Souza, J. L. P. , … Magnusson, W. E. (2012). How far can we go in simplifying biomonitoring assessments? An integrated analysis of taxonomic surrogacy, taxonomic sufficiency and numerical resolution in a megadiverse region. Ecological Indicators, 23, 366–373. 10.1016/j.ecolind.2012.04.023

[ece34284-bib-0093] Lawton, J. H. (1983). Plant architecture and the diversity of phytophagous insects. Annual Review of Entomology, 28, 23–39. 10.1146/annurev.en.28.010183.000323

[ece34284-bib-0094] Lawton, J. H. , & Price, P. W. (1979). Species richness of parasites on hosts: Agromyzid flies on the British Umbelliferae. Journal of Animal Ecology, 48, 619–637. 10.2307/4183

[ece34284-bib-0095] Li, G. , Zhang, S. , & Bater, (2005). Notes of Saihanwula Nature Reserve. Chifeng, China: Science and Technology Press of Inner Mongolia.

[ece34284-bib-0096] Li, G. , Zhang, S. , & Han, L. (1998). Comprehensive Scientific Investigation Reports on Saihanwula National Nature Reserve. Chifeng, China: Saihanwula National Nature Reserve.

[ece34284-bib-0097] Liao, C. , Liu, P. , Xu, J. , Staines, C. L. , & Dai, X. (2018). Description of the last‐instar larva and pupa of a leaf‐mining hispine – *Prionispa champaka* Maulik, 1919 (Coleoptera, Chrysomelidae, Cassidinae, Oncocephalini). ZooKeys, 729, 47–60. 10.3897/zookeys.729.21041 PMC5799732 29416391

[ece34284-bib-0098] Lie, K. J. (1973). Larval trematode antagonism: Principles and possible application as a control method. Experimental Parasitology, 33, 343–349. 10.1016/0014-4894(73)90038-6 4706117

[ece34284-bib-0099] Liu, W. , Dai, X. , & Xu, J. (2015). Influences of leaf‐mining insects on their host plants: A review. Collectanea Botanica, 34, e005.

[ece34284-bib-0100] Locke, S. A. , Mclaughlin, J. D. , & Marcogliese, D. J. (2013). Predicting the similarity of parasite communities in freshwater fishes using the phylogeny, ecology and proximity of hosts. Oikos, 122, 73–83. 10.1111/j.1600-0706.2012.20211.x

[ece34284-bib-0101] Lopez‐Vaamonde, C. , Godfray, H. C. J. , & Cook, J. M. (2003). Evolutionary dynamics of host‐plant use in a genus of leaf‐mining moths. Evolution, 57, 1804–1821. 10.1111/j.0014-3820.2003.tb00588.x 14503622

[ece34284-bib-0102] Lotze, H. K. , & Schramm, W. (2000). Ecophysiological traits explain species dominance patterns in macroalgal blooms. Journal of Phycology, 36, 287–295.

[ece34284-bib-0103] Lotze, H. K. , Worm, B. , & Sommer, U. (2000). Propagule banks, herbivory and nutrient supply control population development and dominance patterns in macroalgal blooms. Oikos, 89, 46–58. 10.1034/j.1600-0706.2000.890106.x

[ece34284-bib-0104] MacArthur, R. H. , & Wilson, E. O. (1967). The theory of island biogeography. Princeton, NJ: Princeton University Press.

[ece34284-bib-0105] MacKenzie, D. I. , & Nichols, J. D. (2004). Occupancy as a surrogate for abundance estimation. Animal Biodiversity and Conservation, 27, 461–467.

[ece34284-bib-0106] McNaughton, S. J. , & Wolf, L. L. (1970). Dominance and niche in ecological systems. Science, 167, 131–139. 10.1126/science.167.3915.131 5409637

[ece34284-bib-0107] Melo, A. S. (2005). Effects of taxonomic and numeric resolution on the ability to detect ecological patterns at a local scale using stream macroinvertebrates. Archiv für Hydrobiologie, 164, 309–323. 10.1127/0003-9136/2005/0164-0309

[ece34284-bib-0108] Mendonça, M. D. S. (2007). Plant diversity and galling arthropod diversity searching for taxonomic patterns in an animal‐plant interaction in the Neotropics. Boletín de la Sociedad Argentina de Botánica, 42, 347–357.

[ece34284-bib-0109] Miller, Z. J. (2012). Fungal pathogen species richness: Why do some plant species have more pathogens than others? American Naturalist, 179, 282–292. 10.1086/663676 22218316

[ece34284-bib-0110] Miller, T. W. , Brodeur, R. D. , Rau, G. , & Omori, K. (2010). Prey dominance shapes trophic structure of the northern California Current pelagic food web: Evidence from stable isotopes and diet analysis. Marine Ecology Progress Series, 420, 15–26. 10.3354/meps08876

[ece34284-bib-0111] Mokany, K. , Ash, J. , & Roxburgh, S. (2008). Functional identity is more important than diversity in influencing ecosystem processes in a temperate native grassland. Journal of Ecology, 96, 884–893. 10.1111/j.1365-2745.2008.01395.x

[ece34284-bib-0112] Montgomery, W. L. (1980). The impact of non‐selective grazing by the giant blue damselfish, Microspathodon on algal communities in the gulf of California, Mexico. Bulletin of Marine Science, 30, 290–303.

[ece34284-bib-0113] Mori, S. A. , Boom, B. M. , de Carvalino, A. M. , & dos Santos, T. S. (1983). Ecological importance of Myrtaceae in an Eastern Brazilian wet forest. Biotropica, 15, 68–70. 10.2307/2388002

[ece34284-bib-0114] Mouquet, N. , Gravel, D. , Massol, F. , & Calcagno, V. (2013). Extending the concept of keystone species to communities and ecosystems. Ecology Letters, 16, 1–8. 10.1111/ele.12014 23062191

[ece34284-bib-0115] Nakamura, T. , Hattori, K. , Ishida, T. A. , Sato, H. , & Kimura, M. T. (2008). Population dynamics of leafminers on a deciduous oak *Quercus dentata* . Acta Oecologica, 34, 259–265. 10.1016/j.actao.2008.03.008

[ece34284-bib-0116] Okullo, P. , Greve, P. M. K. , & Moe, S. R. (2013). Termites, large herbivores, and herbaceous plant dominance structure small mammal communities in savannahs. Ecosystems, 16, 1002–1012. 10.1007/s10021-013-9663-2

[ece34284-bib-0117] Olff, H. , & Ritchie, M. E. (1998). Effect of herbivores on grassland plant diversity. Trends in Ecology & Evolution, 13, 261–265. 10.1016/S0169-5347(98)01364-0 21238294

[ece34284-bib-0118] O'Meara, B. C. , Graham, K. L. , Pellis, S. M. , & Burghardt, G. M. (2015). Evolutionary models for the retention of adult–adult social play in primates: The roles of diet and other factors associated with resource acquisition. Adaptive Behavior, 23, 381–391. 10.1177/1059712315611733

[ece34284-bib-0119] Opler, P. A. (1974). Oaks as evolutionary islands for leaf‐mining insects: the evolution and extinction of phytophagous insects is determined by an ecological balance between species diversity and area of host occupation. American Scientist, 62, 67–73.

[ece34284-bib-0120] Opler, P. A. , & Davis, D. R. (1981). The leafmining moths of the genus *Cameraria* associated with Fagaceae in California (Lepidoptera: Gracillariidae). Smithsonian Contributions to Zoology, 333, 1–58. 10.5479/si.00810282.333

[ece34284-bib-0121] Paradis, E. , & Claude, J. (2002). Analysis of comparative data using generalized estimating equations. Journal of Theoretical Biology, 218, 175–185. 10.1006/jtbi.2002.3066 12381290

[ece34284-bib-0122] Paradis, E. , Claude, J. , & Strimmer, K. (2004). APE: Analyses of phylogenetics and evolution in R language. Bioinformatics, 20, 289–290. 10.1093/bioinformatics/btg412 14734327

[ece34284-bib-0123] Perry, N. (2010). The ecological importance of species and the Noah's Ark problem. Ecological Economics, 69, 478–485. 10.1016/j.ecolecon.2009.09.016

[ece34284-bib-0124] Phillips, O. , & Gentry, A. H. (1993). The useful plants of Tambopata, Peru: II. Additional hypothesis testing in quantitative ethnobotany. Economic Botany, 47, 33–43. 10.1007/BF02862204

[ece34284-bib-0125] Pierce, S. , Luzzaro, A. , Caccianiga, M. , Ceriani, R. M. , & Cerabolini, B. (2007). Disturbance is the principal α‐scale filter determining niche differentiation, coexistence and biodiversity in an alpine community. Journal of Ecology, 95, 698–706. 10.1111/j.1365-2745.2007.01242.x

[ece34284-bib-0126] Poulin, R. (2010). Decay of similarity with host phylogenetic distance in parasite faunas. Parasitology, 137, 733–741. 10.1017/S0031182009991491 19849890

[ece34284-bib-0127] Poulin, R. , & Krasnov, B. (2010). Similarity and variability of parasite assemblages across geographical space In Morand S., & Krasnov B. R. (Eds.), The biogeography of Host‐Parasite interactions (pp. 115–127). New York, NY: Oxford University Press.

[ece34284-bib-0128] Poulos, H. M. , Taylor, A. H. , & Beaty, R. M. (2007). Environmental controls on dominance and diversity of woody plant species in a Madrean, Sky Island ecosystem, Arizona, USA. Plant Ecology, 193, 15–30. 10.1007/s11258-006-9245-x

[ece34284-bib-0129] Power, M. E. , Tilman, D. , Estes, J. A. , Menge, B. A. , Bond, W. J. , Mills, L. S. , … Paine, R. T. (1996). Challenges in the quest for keystones: Identifying keystone species is difficult—but essential to understanding how loss of species will affect ecosystems. BioScience, 46, 609–620. 10.2307/1312990

[ece34284-bib-0130] Price, P. W. (1977). General concepts on the evolutionary biology of parasites. Evolution, 31, 405–420. 10.1111/j.1558-5646.1977.tb01021.x 28563224

[ece34284-bib-0131] R Core Team . (2018). R: A language and environment for statistical computing. Vienna, Austria: R Foundation for Statistical Computing.

[ece34284-bib-0132] Ribas, L. G. S. , & Padial, A. A. (2015). The use of coarser data is an effective strategy for biological assessments. Hydrobiologia, 747, 83–95. 10.1007/s10750-014-2128-6

[ece34284-bib-0133] Roth, G. A. , Whitford, W. G. , & Steinberger, Y. (2007). Jackrabbit (*Lepus californicus*) herbivory changes dominance in desertified Chihuahuan Desert ecosystems. Journal of Arid Environments, 70, 418–426. 10.1016/j.jaridenv.2007.01.009

[ece34284-bib-0134] RStudio Team . (2018). RStudio: Integrated development for R. Boston, MA: RStudio Inc.

[ece34284-bib-0135] Santamaria, L. (2002). Selective waterfowl herbivory affects species dominance in a submerged plant community. Archiv für Hydrobiologie, 153, 353–365. 10.1127/archiv-hydrobiol/153/2002/353

[ece34284-bib-0136] Sato, H. (1991). Differential resource utilization and co‐occurrence of leaf miners on oak (*Quercus dentata*). Ecological Entomology, 16, 105–113. 10.1111/(ISSN)1365-2311

[ece34284-bib-0137] Schlinkert, H. , Westphal, C. , Clough, Y. , László, Z. , Ludwig, M. , & Tscharntke, T. (2015). Plant size as determinant of species richness of herbivores, natural enemies and pollinators across 21 Brassicaceae species. PLoS ONE, 10, e0135928 10.1371/journal.pone.0135928 26291614 PMC4546192

[ece34284-bib-0138] Schmook, B. (2010). Shifting maize cultivation and secondary vegetation in the Southern Yucatán: Successional forest impacts of temporal intensification. Regional Environmental Change, 10, 233–246. 10.1007/s10113-010-0128-2

[ece34284-bib-0139] Schweiger, A. H. , & Beierkuhnlein, C. (2014). Water temperature and acidity regime shape dominance and beta‐diversity patterns in the plant communities of springs. Frontiers of Biogeography, 6, 132–143.

[ece34284-bib-0140] Seabloom, E. W. , Borer, E. T. , Buckley, Y. M. , Cleland, E. E. , Davies, K. F. , Firn, J. , … Yang, L. (2015). Plant species' origin predicts dominance and response to nutrient enrichment and herbivores in global grasslands. Nature Communications, 6, 7710 10.1038/ncomms8710 PMC4518311 26173623

[ece34284-bib-0141] Seifertová, M. , Vyskočilová, M. , Morand, S. , & Šimková, A. (2008). Metazoan parasites of freshwater cyprinid fish (*Leuciscus cephalus*): Testing biogeographical hypotheses of species diversity. Parasitology, 135, 1417–1435. 10.1017/S0031182008004812 18775091

[ece34284-bib-0142] Sinclair, R. J. , & Hughes, L. (2008a). Incidence of leaf mining in different vegetation types across rainfall, canopy cover and latitudinal gradients. Austral Ecology, 33, 353–360. 10.1111/j.1442-9993.2007.01825.x

[ece34284-bib-0143] Sinclair, R. J. , & Hughes, L. (2008b). Leaf mining in the Myrtaceae. Ecological Entomology, 33, 623–630. 10.1111/j.1365-2311.2008.01014.x

[ece34284-bib-0144] Smilanich, A. M. , Fincher, R. M. , & Dyer, L. A. (2016). Does plant apparency matter? Thirty years of data provide limited support but reveal clear patterns of the effects of plant chemistry on herbivores. New Phytologist, 210, 1044–1057. 10.1111/nph.13875 26889654

[ece34284-bib-0145] Smith, L. M. , Blue, J. , Carlson, J. , Metz, G. , Haywood, J. , Bush, D. , & Paradise, C. J. (2009). Density‐dependent predation of a dominant species does not facilitate increased diversity in treeholes. The Open Ecology Journal, 2, 91–99. 10.2174/1874213000902010091

[ece34284-bib-0146] Smith, R. , & Smith, T. (2001). Ecology and field biology, 6th edition. San Francisco, CA: Benjamin Cummings Inc.

[ece34284-bib-0147] Soldati, G. T. , de Medeiros, P. M. , Duque‐Brasil, R. , Coelho, F. M. G. , & Albuquerque, U. P. (2017). How do people select plants for use? Matching the Ecological Apparency Hypothesis with Optimal Foraging Theory. Environment, Development and Sustainability, 19, 2143–2161. 10.1007/s10668-016-9844-1

[ece34284-bib-0148] Strona, G. , & Fattorini, S. (2014). A few good reasons why species‐area relationships do not work for parasites. BioMed Research International, 2014, 271680. 24895561 10.1155/2014/271680 PMC4034449

[ece34284-bib-0149] Sugiura, S. (2010). Associations of leaf miners and leaf gallers with island plants of different residency histories. Journal of Biogeography, 37, 237–244. 10.1111/j.1365-2699.2009.02199.x

[ece34284-bib-0150] Takemoto, K. , & Aie, K. (2017). Limitations of a metabolic network‐based reverse ecology method for inferring host–pathogen interactions. BMC Bioinformatics, 18, 278 10.1186/s12859-017-1696-7 28545448 PMC5445277

[ece34284-bib-0151] Thomas, E. , Vandebroek, I. , & Van Damme, P. (2009). Valuation of forests and plant species in indigenous territory and national park Isiboro‐Sécure, Bolivia. Economic Botany, 63, 229–241. 10.1007/s12231-009-9084-5

[ece34284-bib-0152] Tweedley, J. R. , Warwick, R. M. , & Potter, I. C. (2015). Can biotic indicators distinguish between natural and anthropogenic environmental stress in estuaries? Journal of Sea Research, 102, 10–21. 10.1016/j.seares.2015.04.001

[ece34284-bib-0153] Veldtman, R. , & McGeoch, M. A. (2003). Gall‐forming insect species richness along a non‐scleromorphic vegetation rainfall gradient in South Africa: The importance of plant community composition. Austral Ecology, 28, 1–13. 10.1046/j.1442-9993.2003.01234.x

[ece34284-bib-0154] Ward, L. K. , & Spalding, D. F. (1993). Phytophagous British insects and mites and their food‐plant families: Total numbers and polyphagy. Biological Journal of the Linnean Society, 49, 257–276. 10.1111/j.1095-8312.1993.tb00905.x

[ece34284-bib-0155] Webb, C. , Ackerly, D. , & Kembel, S. (2008). Phylocom: Software for the analysis of phylogenetic community structure and character evolution. Bioinformatics, 24, 2098–2100. 18678590 10.1093/bioinformatics/btn358

[ece34284-bib-0156] Webb, C. O. , & Donoghue, M. J. (2005). Phylomatic: Tree assembly for applied phylogenetics. Molecular Ecology Notes, 5, 181–183. 10.1111/j.1471-8286.2004.00829.x

[ece34284-bib-0157] Whittaker, R. H. (1965). Dominance and diversity in land plant communities : Numerical relations of species express the importance of competition in community function and evolution. Science, 147, 250–260. 10.1126/science.147.3655.250 17788203

[ece34284-bib-0158] Wu, L. , Shinzato, T. , Kudo, T. , Ishigaki, C. , & Aramoto, M. (2008). Characteristics of a 20‐year‐old evergreen broad‐leaved forest restocked by natural regeneration after clearcut‐burning. Annals of Forest Science, 65, 505–505. 10.1051/forest:2008031

[ece34284-bib-0159] Xu, J. , Dai, X. , Liao, C. , Diškus, A. , & Stonis, J. R. (2018). Discovery of Ulmaceae‐feeding Tischeriidae (Lepidoptera, Tischerioidea), *Tischeria ulmella* sp. nov., and the first report of the Quercus‐feeding *T. naraensis* Sato in China. Zootaxa, 4399, 361–370. 10.11646/zootaxa.4399.3.6 29690319

[ece34284-bib-0160] Xu, J. , Dai, X. , Liu, P. , Bai, H. , Dikus, A. , & Stonis, J. R. (2017). First report on Paratischeria from Asia (Lepidoptera: Tischeriidae). Zootaxa, 4350, 331–344. 10.11646/zootaxa.4350.2.8 29245557

[ece34284-bib-0161] Yu, F. , Shi, X. , Wang, D. , Wang, T. , Yi, X. , & Lou, Y. (2014). Seed predation patterns favor the regeneration of dominant species in forest gaps compared with the understory in an oak‐pine mixed forest. Acta Theriologica, 59, 495–502. 10.1007/s13364-014-0192-y

[ece34284-bib-0162] Yu, Q. , Wilcox, K. , La Pierre, K. , Knapp, A. K. , Han, X. , & Smith, M. D. (2015). Stoichiometric homeostasis predicts plant species dominance, temporal stability and responses to global change. Ecology, 96, 2328–2335. 10.1890/14-1897.1 26594691

[ece34284-bib-0163] Zhang, S. (2007). Study on plants diversity and conservation in Saihanwula Nature Reserve, Inner Mongolia. (Doctoral Dissertation). Beijing, China: Beijing Forestry University.

[ece34284-bib-0164] Zhang, J. (2017). plantlist: Looking up the status of plant scientific names based on the plant list database. R package version 0.3.0. https://github.com/helixcn/plantlist/

[ece34284-bib-0165] Zheng, H. , Gao, J. , Teng, Y. , Feng, C. , & Tian, M. (2015). Temporal variations in soil moisture for three typical vegetation types in Inner Mongolia, Northern China. PLoS One, 10, e0118964 10.1371/journal.pone.0118964 25781333 PMC4363572

